# A novel method to estimate the absorption rate constant for two-compartment model fitted drugs without intravenous pharmacokinetic data

**DOI:** 10.3389/fphar.2023.1087913

**Published:** 2023-05-04

**Authors:** Fan Liu, Hanxi Yi, Lei Wang, Zeneng Cheng, Guoqing Zhang

**Affiliations:** ^1^ Xiangya School of Pharmaceutical Sciences, Central South University, Changsha, Hunan, China; ^2^ Department of Neurology, Xiangya Hospital, Central South University, Changsha, Hunan, China; ^3^ School of Basic Medicine, Central South University, Changsha, Hunan, China; ^4^ Department of Rheumatology and Immunology, The Second Clinical Medical College, Jinan University (Shenzhen People's Hospital), Shenzhen, China; ^5^ Integrated Chinese and Western Medicine Postdoctoral Research Station, Jinan University, Guangzhou, China

**Keywords:** absorption rate constant, the direct method, maximum apparent rate constant of disposition, two-compartment model, extravascular administration

## Abstract

The *in vivo* performances of most drugs after extravascular administration are fitted well with the two-compartment pharmacokinetic (PK) model, but the estimation of absorption rate constant (k_a_) for these drugs becomes difficult during unavailability of intravenous PK data. Herein, we developed a novel method, called the direct method, for estimating the k_a_ values of drugs without using intravenous PK data, by proposing a new PK parameter, namely, maximum apparent rate constant of disposition (k_max_). The accuracy of the direct method in k_a_ estimation was determined using the setting parameters (k_12_, k_21_, and k_10_ values at high, medium, and low levels, respectively) and clinical data. The results showed that the absolute relative error of k_a_ estimated using the direct method was significantly lower than that obtained using both the Loo-Riegelman method and the statistical moment method for the setting parameters. Human PK studies of telmisartan, candesartan cilexetil, and tenofovir disoproxil fumarate indicated that the k_a_ values of these drugs were accurately estimated using the direct method based on good correlations between the k_a_ values and other PK parameters that reflected the absorption properties of drugs *in vivo* (T_max_, C_max_, and C_max_/AUC_0-t_). This novel method can be applied in situations where intravenous PK data cannot be obtained and is expected to provide valuable support for PK evaluation and in vitro-in vivo correlation establishment.

## 1 Introduction

The absorption rate of drugs refers to the rate at which the drug enters systemic circulation after passing through the mucosal lining since extravascular administration (i.e., orally, perorally, rectally, etc.), and this rate consequently affects the peak time (T_max_) and peak concentration (C_max_) of drugs *in vivo* (Tozer et al., 1996). Quantitative assessment of the drug absorption rate constant (k_a_) plays a vital role in the pharmaceutical industry. For instance, the correlation between the *in vivo* absorption rate and the *in vitro* dissolution rate (IVIVC) of a dosage form can predict the bioavailability of a drug and help avoid excessive number of clinical trials (Zhang et al., 2021). According to the U.S. Food and Drug Administration (FDA), proprietary preparations with identical active pharmaceutical ingredients are regarded as bioequivalents if the rate and extent of drug absorption between the test and reference formulations do not show any significant differences (FDA, 2003). To date, several methods have been widely employed for k_a_ estimation, and can be classified into two different categories: i) methods based on the compartmental pharmacokinetic (PK) model, including the Wagner-Nelson method (suitable for the one-compartment PK model) and the Loo-Riegelman method (suitable for the two-compartment PK model); ii) methods based on the non-compartmental PK model, including the numerical deconvolution method and the statistical moment method.

**FIGURE 1:**
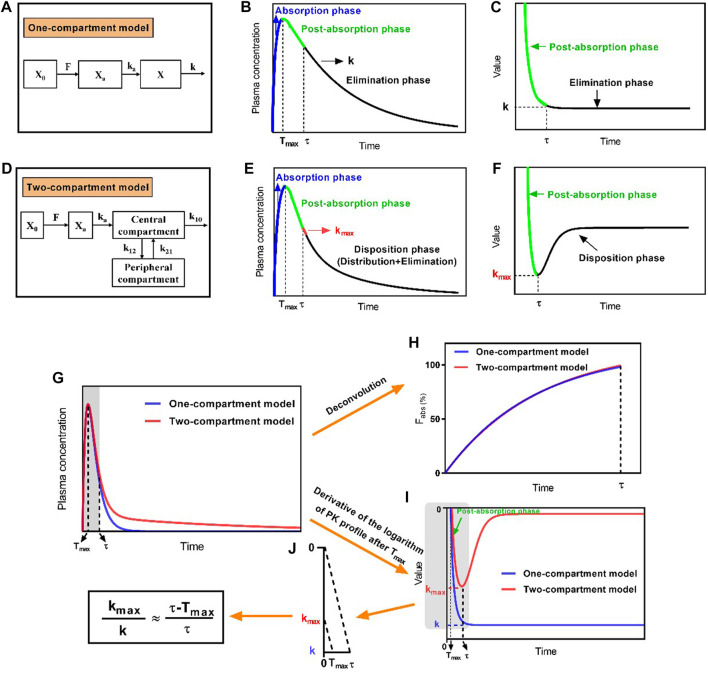
Schematic diagram of the development of the direct method by proposing the maximum apparent rate constant of disposition (k_max_) and its corresponding time point (τ) in the two-compartment model. **(A)** Schematic diagram of the extravascular administration for the one-compartment model, **(B)** characteristic profile of the one-compartment model, and **(C)** derivative of the logarithmic plasma drug concentration–time profile after T_max_, which shows an invariable elimination rate constant (k); **(D)** schematic diagram of the extravascular administration for the two-compartment model, **(E)** characteristic profile of the two-compartment model, and **(F)** derivative of the logarithmic plasma drug concentration–time profile after T_max_, for which the k_max_ and its corresponding time point of τ were available; **(G)** plasma drug concentration–time profile of drugs fitting the one-compartment model or two-compartment model; **(H)** absorption profiles of drugs after deconvolution; **(I)** derivative of the logarithmic plasma drug concentration–time profiles; **(J)** The relationship of $\frac{k_{\max}}{k}\approx \frac{\tau -T_{\max}}{\tau }$ .

In addition to the absorption and elimination phases, the two-compartment model for a drug includes a distribution phase, where the drug is distributed from a central compartment to a peripheral compartment; this model differs from the one-compartment model that treats the body as one uniform component (Figures 1A, D). In this case, the Loo-Riegelman method is the classic method, as it considers the distribution phase for estimating the k_a_ values of drugs with the two-compartment model. This method requires the data of PK parameters including k_10_ (first-order elimination rate constant), k_12_ (first-order rate constant of the drugs transfer from the central compartment to the peripheral compartment), and k_21_ (first-order rate constant of the drugs transfer from the peripheral compartment to the central compartment); these data need to be obtained from the intravenous administration of the corresponding drugs to estimate their k_a_ (Wagner, 1975). The numerical deconvolution method calculates the k_a_ of drugs and does not involve the limitations of the compartmental model, but it requires the same sampling time and intervals for both intravenous and extravascular administrations (Yu et al., 1996). Thus, intravenous PK data are necessary for estimating the k_a_ when using either the Loo-Riegelman method or the numerical deconvolution method. However, determining the intravenous PK parameters of drugs is challenging if they can be administered only through the extravascular route because of safety concerns in human volunteers.

The statistical moment method can also be applied to the non-compartmental PK model by applying overall random variables obtained from the *in vivo* process of drugs. k_a_ is estimated by calculating the difference in mean residence time (MRT) between various types of administrations to avoid the use of intravenous PK data. However, many factors affect the accuracy of k_a_ estimated using the statistical moment method, such as the precision of detecting low plasma drug concentration and the lack of appropriate data for determining the logarithmic linearity in the terminal phase that yields the accurate elimination rate constant (k_T_) (Riegelman and Collier, 1980). Therefore, the deficiency in intravenous PK data or poor accuracy of the method hinders k_a_ estimation for drugs with the two-compartment model.

Generally, the plasma concentration (C) and k_a_ of drugs for extravascular administration in the one-compartment model had the following relationship (Eq. 1):

$$C=\frac{k_{a}FX_{0}}{V\left(k_{a}-k\right)}\exp\left(-kt\right)-\frac{k_{a}FX_{0}}{V\left(k_{a}-k\right)}\exp\left(-k_{a}t\right)$$ (1)

where F is the drug bioavailability, X_0_ is the dose, V is the apparent volume of distribution, and k is the elimination rate constant. When differentiating with respect to time t, it gets the following equation:

$$\frac{dC}{dt}=\frac{k_{a}^{2}FX_{0}}{V\left(k_{a}-k\right)}\exp\left(-k_{a}t\right)-\frac{k_{a}kFX_{0}}{V\left(k_{a}-k\right)}\exp\left(-kt\right)$$ (2)

As the plasma drug concentration reached the C_max_ (i.e., $\frac{dC}{dt}=0$ ), Eq. 2 was simplified to Eq. 3, which was a classical equation to quickly calculate k_a_ for the one-compartment model (Zhi, 1990).

$$T_{\max}=\frac{\ln k_{a}-lnk}{k_{a}-k}$$ (3)

When the PK model was not considered, the concentration–time curve consisted of two sections: the first-order rate increase curve and the first-order rate decrease curve. The basic formula satisfied $C=Aexp\left(-kt\right)-Bexp\left(-k_{a}t\right)$ , where k is the elimination rate constant in the one-compartment model or the total removal rate constant of the drugs removed from the central compartment because of their distribution (k_12_) and elimination (k_10_) in the two-compartment model. Thus, k_a_ was estimated for drugs that fitted with the two-compartment model after the k in Eq. 3 was replaced with “k_12_ + k_10_,” referred to as the alternative method (Zeng et al., 2020). This method has excellent accuracy and convenience compared with both the Loo-Riegelman method and the statistical moment method. However, the alternative method also requires intravenous PK data to calculate k_10_ and k_12_. Thus, identifying a novel PK parameter in the two-compartment model to replace the k (in Eq. 3) may be one of the effective ways for estimating k_a_ without the need for intravenous PK data.

In the present study, a new parameter, namely, maximum apparent rate constant of disposition (k_max_), was defined to develop a novel method (named as “the direct method”) for k_a_ estimation. The accuracy of k_a_ estimated using the direct method was investigated by setting the k_12_, k_21_, and k_10_ values at high, medium, and low levels, respectively, after the relationship and range of these parameters were determined from previously published reports. Additionally, the accuracy of the k_a_ value estimated using the direct method was compared with the accuracies determined using the Loo-Riegelman method and the statistical moment method. Three model drugs (telmisartan (TMS), candesartan cilexetil (CSC), and tenofovir disoproxil fumarate (TDF)) with different formulations were selected, and their PK parameters were assessed in humans. The direct method was used to estimate the k_a_ values of three model drugs, and from the results, correlations were established between their estimated k_a_ values and the other PK parameters that reflected the absorption properties of the drugs *in vivo*. These correlations were analyzed to verify the accuracy of the direct method in estimating the k_a_ value of drugs.

## 2 Materials and methods

### 2.1 Materials

Tablet dosage forms with different immediate-release (IR) formulations, including TMS (F_M1_ and F_M2_, specifications: 80 mg), CSC (F_C1_ and F_C2_, specifications: 4 mg), and TDF (F_D1_ and F_D2_, specifications: 300 mg), were kindly supplied by three different pharmaceutical companies.

### 2.2 Development of the direct method for k_a_ estimation

#### 2.2.1 Definition of k_max_

Unlike the one-compartment model, which has an invariable value of k (Figure 1B), the plasma drug concentration–time curve that fixed well with the two-compartment model was divided into three phases: the absorption phase, post-absorption phase, and disposition phase (i.e., sum of the distribution and elimination phase; Figure 1E). The portion of the curve before T_max_ represented the absorption phase, during which the rate of increasing plasma drug concentration was significantly higher than the rate of its disposition, and the portion of the curve after T_max_ represented the post-absorption phase, during which the disposition rates of the drugs were higher than the absorption rates. Thereafter, the disposition rate gradually decreased until it reached an invariable terminal elimination process. At the end time of the post-absorption phase (τ), the absorption phase had completed; thus, only the disposition phase remained. This phase presented the highest apparent rate of drug disposition (k_max_) at the first time interval after τ (Figure 1E). Moreover, the derivative of the logarithm of the plasma drug concentration–time profile reflected the real-time rate of decreasing drug concentration (i.e., the slope of the logarithmic PK curve after T_max_), which gradually increased and then remained at a constant rate (k) for the one-compartment model because of the presence of the post-absorption phase after T_max_ (Figure 1C). By contrast, the rate of declining drug concentration continuously showed changes in the order of increase, decrease, and constant that presented the k_max_ at τ for the two-compartment model (Figure 1F).

#### 2.2.2 Development of the direct method

The k_a_, X_0_, F, and V in the one-compartment model and two-compartment model were set as the same values, as well as k = k_12_ + k_10_. The absorption phase, post-absorption phase, and disposition phase satisfied first-order kinetics. The absorption phases of two simulated drug concentration–time curves had almost overlapped (Figure 1G). The absorption profiles had also overlapped after deconvolution (Figure 1H). The absorption was complete at time point τ, which corresponded to k_max_. After the derivative of the logarithmic plasma drug concentration–time profile, k_max_ and k showed unequal values, and the value of k_max_ was always less than that of k, but the value of τ was always greater than that of T_max_. When the values of k_max_, k, T_max_, and τ were extracted from Figure 1I, the four parameters had the following relationship after proportional scaling of triangles (Eq. 4; Figure 1J).

$$\frac{k_{\max}}{k}\approx \frac{\tau -T_{\max}}{\tau }$$ (4)

Equation 4 was transformed into Eq. 5:

$$k\approx \frac{{\tau *k}_{\max}}{\tau -T_{\max}}$$ (5)

Thus, Eq. 3 was approximately transformed into Eq. 6 using the relationship established in Eq. 5.

$$T_{\max}=\frac{\ln k_{a}-\ln\frac{{\tau *k}_{\max}}{\tau -T_{\max}}}{k_{a}-\frac{{\tau *k}_{\max}}{\tau -T_{\max}}}$$ (6)

In this case, the values of T_max_ were obtained from the plasma drug concentration–time curves, and the values of k_max_ and τ were obtained from the logarithm of the plasma drug concentration–time curves for the two-compartment model after extravascular administration. Subsequently, k_a_ was estimated using Newton’s iteration method with the Python software package (version 3.6.7). Therefore, the direct method did not require measurement of the intravenous concentration of drugs.

### 2.3 Validation of the direct method by setting parameters

#### 2.3.1 Parameter setting and model judgment

To ensure that the setting parameters satisfied the two-compartment model, the human plasma drug concentration–time curves of 36 drugs fitting the two-compartment model in the fasted or fed states were obtained from previously published reports, and the corresponding data were extracted using GetData Graph Digitizer software (version 2.25, https://www.getdata-graph-digitizer.com/). The preliminary k_a_, k_12_, k_21_, and k_10_ values of these drugs were calculated using WinNonlin software (version 8.2, Certara Co., United States), which were attributed to the inability to obtain these parameters from the literature.

The k_a_, k_12_, k_21_, and k_10_ values were sorted in the descending order. The average values of the top one-third, middle one-third, and bottom one-third of these data (*n* = 12) were set as high, medium, and low levels, respectively. Then, the different levels of each parameter were combined randomly. Plasma drug concentration was calculated at different time points (intervals of 0.1 h) after factoring the setting parameters (k_a_, k_12_, k_21_, and k_10_) into the following Eqs 7–9:

$$C=\frac{k_{a}FX_{0}\left(k_{21}-k_{a}\right)}{V_{c}\left(\alpha -k_{a}\right)\left(\beta -k_{a}\right)}∙\exp\left(-k_{a}t\right)+\frac{k_{a}FX_{0}\left(k_{21}-\alpha \right)}{V_{c}\left(k_{a}-\alpha \right)\left(\beta -\alpha \right)}∙\exp\left(-\alpha t\right)+\frac{k_{a}FX_{0}\left(k_{21}-\beta \right)}{V_{c}\left(k_{a}-\beta \right)\left(\alpha -\beta \right)}∙\exp\left(-\beta t\right)$$ (7)

where X_0_, F, and V_c_ were randomly set as fixed values (e.g., X_0_ = 2,200 μg, F = 1, V_c_ = 10 L). The α and β variables in Eq. 7, which represent the distribution phase mixed first-order rate constant and the elimination phase mixed first-order rate constant, respectively, were determined using Eqs 8, 9:

$$\alpha =\frac{\left(k_{12}{+k}_{21}{+k}_{10}\right)+\sqrt{{\left(k_{12}{+k}_{21}{+k}_{10}\right)}^{2}-4k_{21}k_{10}}}{2}$$ (8)

$$\beta =\frac{\left(k_{12}{+k}_{21}{+k}_{10}\right)-\sqrt{{\left(k_{12}{+k}_{21}{+k}_{10}\right)}^{2}-4k_{21}k_{10}}}{2}$$ (9)

Furthermore, the Akaike information criteria (AIC) values were calculated using Eqs 10, 11 to evaluate the compartmental model of the drug concentration–time curves.

$$AIC=N∙\ln R_{e}+2p$$ (10)

$$R_{e}=\sum_{i-1}^{n}W_{i}{\left(C_{i}-{\hat{C}}_{i}\right)}^{2}$$ (11)

where N is the number of experimental groups, R_e_ is the sum of squares of the weighted residuals, p is the number of model parameters, W_i_ is the weight coefficient, C_i_ is the experimental plasma drug concentration, and Ĉ_i_ is the estimated plasma drug concentration. The AIC values of drugs in the one-compartment model and two-compartment model were calculated; the smaller the AIC value, the better the fitting (Kadam et al., 2013).

#### 2.3.2 Estimation of k_a_ using the direct method

T_max_ was determined from the data of the plasma drug concentration–time curves of the setting parameters. The k_max_ was fitted from the slope of the logarithm of plasma drug concentration–time curve at the first time interval after the time point τ. The k_a_ value was then estimated using the direct method (Eq. 6). The accuracy of k_a_ estimation was calculated by comparing the estimated k_a_ from Eq. 6 to the setting value of k_a_ (i.e., the true k_a_ value) using Eq. 12:

$$Relative\;error\;\left(RE\right)\%=\frac{{k_{a\left(estimation\right)}-k}_{a\left(true\right)}}{k_{a\left(true\right)}}\times 100\%$$ (12)

#### 2.3.3 Estimation of k_a_ using the Loo-Riegelman method

The setting k_12_, k_21_, and k_10_ values were used to estimate the k_a_ value using the Loo-Riegelman method. Briefly, k_a_ was calculated using the following equation (Eq. 13):

$$\ln\left(1-F_{abs}\right)=-k_{a}t+b$$ (13)

and the *in vivo* absorption fraction (F_abs_) was obtained using Eq. 14:

$$F_{abs}=\frac{{\left(X_{A}\right)}_{t}}{{\left(X_{A}\right)}_{\infty }}=\frac{C_{t}+k_{10}\int_{0}^{t}Cd_{t}+\frac{{\left(X_{p}\right)}_{t}}{V_{c}}}{k_{10}\int_{0}^{\infty }Cd_{t}}$$ (14)

The $\frac{{\left(X_{p}\right)}_{t}}{V_{c}}$ value in Eq. 14 was calculated using Eq. 15:

$$\frac{{\left(X_{p}\right)}_{t}}{V_{c}}=\frac{{\left(X_{p}\right)}_{t-1}}{V_{C}}\exp\left(-k_{21}∆t\right)+\frac{k_{12}C_{0}}{k_{21}}\;\left[1-\exp\left(-k_{21}∆t\right)\right]+\frac{k_{12}\left(\frac{∆c}{∆t}\right)\;{∆t}^{2}}{2}$$ (15)

where ${\left(X_{A}\right)}_{t}$ and ${\left(X_{A}\right)}_{\infty }$ are the amount of drug entering systemic circulation at time t and infinite time, respectively. ${\left(X_{p}\right)}_{t}$ is the amount of drug entering the peripheral compartment at time t. Moreover, ∆c and ∆t represent the differences in the plasma drug concentration and time between two consecutive samples, respectively.

#### 2.3.4 Estimation of k_a_ using the statistical moment method

The k_a_ value determined upon fitting the plasma drug concentration–time data of the setting parameters with the statistical moment method was compared with that determined upon fitting plasma drug concentration–time data with the direct method. The calculation of the statistical moment method performed to make this comparison is shown in Eq. 16:

$$\frac{1}{k_{a}}=MAT=MRT-\frac{1}{k_{T}}=\frac{AUMC}{AUC}-\frac{1}{k_{T}}$$ (16)

where MAT is the average absorption time, MRT is the average residence time after extravascular administration, and k_T_ is the elimination rate constant at the terminal phase. The area under the plasma drug concentration–time curve (AUC) was calculated using the trapezoidal method. AUMC, which represented the area under the moment curve, was calculated using Eq. 17:

$$AUMC=\sum_{i=0}^{n-1}\frac{c_{i}t_{i}+c_{i+1}t_{i+1}}{2}\left(t_{i+1}-t_{i}\right)+\frac{c_{n}t_{n}}{k_{T}}+\frac{c_{n}}{{k_{T}}^{2}}$$ (17)

where C_i_, C_i+1_, and C_n_ are the drug concentrations at time points t_i_, t_i+1_, and t_n_, respectively.

### 2.4 Validation of the direct method using clinical data

#### 2.4.1 Clinical data of the model drugs

The plasma concentrations of three model drugs, namely, TMS, CSC, and TDF, were obtained from PK studies involving healthy human volunteers. The clinical studies were conducted in accordance with the Declaration of Helsinki, and the experimental protocols were approved by the Chinese Food and Drug Administration (CFDA) and the Institutional Research Ethics Committee of Xiangya School of Pharmacy, Central South University (project code: 2020006). All enrolled volunteers were fully informed of the protocol of the clinical studies, and their consents to participate were approved. PK studies had randomized, open-label, and single-dose designs, wherein the PK parameters were compared after the oral administration of different formulations containing TMS, CSC, or TDF.

Briefly, PK studies of TMS tablets were conducted with a two-way crossover design on 26 healthy volunteers in the fasted state, which included a 7-day washout period between treatments. Blood samples were collected in heparin-containing vacutainers before administration (0 h) and 0.17, 0.33, 0.5, 0.75, 1, 1.25, 1.5, 2, 2.5, 3, 4, 6, 8, 10, 12, 24, 48, 72, and 96 h after the administration of the F_M1_ or F_M2_ tablets.

PK studies of CSC tablets were conducted with a two-way crossover design on 24 volunteers in the fasted state, which included a 7-day washout period between treatments. Blood samples were collected in heparin-containing vacutainers before administration (0 h) and 0.33, 0.67, 1, 1.33, 1.67, 2, 2.33, 2.67, 3, 4, 6, 8, 12, 24, and 48 h after the administration of the F_C1_ or F_C2_ tablets.

PK studies of TDF tablets were conducted with a two-way crossover design on 24 volunteers in the fasted state and the fed state (the fed state consisted of a high-fat meal with a nutritional composition of 522-kcal fat, 288-kcal carbohydrates, 149-kcal protein, and 959-kcal total calories). Studies of TDF tablets featured the 7-day washout period between treatments. Blood samples were collected in heparin-containing vacutainers before administration (0 h) and 0.25, 0.5, 1, 1.5, 2, 2.5, 3, 3.5, 4, 5, 6, 8, 10, 12, 24, 36, and 48 h after the administration of the F_D1_ or F_D2_ tablets.

All blood samples were centrifuged at 3,500 rpm for 10 min. The plasma samples were separated and then stored at −70°C until analysis by high-performance liquid chromatography-tandem mass spectrometry (Agilent, United States).

#### 2.4.2 Determination of PK parameters

CSC and TDF were rapidly and completely hydrolyzed to candesartan and tenofovir in the plasma, respectively, after absorption from the gastrointestinal tract (Gleiter and Morike, 2002; Kearney et al., 2004). The U.S. FDA recommended the detection of plasma concentrations of candesartan and tenofovir in human PK studies of CSC tablet (FDA, 2008) and TDF tablet (FDA, 2012), respectively. PK parameters, namely, C_max_, T_max_, AUC_0-t_, AUC_0-∞_, and elimination half-life (t_1/2_), of TMS, candesartan, and tenofovir were calculated using the WinNonlin software package. All data were expressed as mean ± standard deviation.

#### 2.4.3 Validation of the direct method

The values of k_max_ and τ for TMS, CSC, and TDF were obtained by calculating the logarithm of the plasma drug concentration–time curves. The k_a_ values for TMS, CSC, and TDF were estimated using the direct method (Eq. 6), statistical moment method (Eq. 16), and Loo-Riegelman method (Eq. 13), respectively. Pearson’s correlation analysis (SPSS 25.0; SPSS Inc., United States) was performed to evaluate the relationship between the k_a_ values and other PK parameters that reflected the absorption properties of the drugs *in vivo* (T_max_, C_max_, and C_max_/AUC_0-t_). Furthermore, the absorption rate *versus* time profiles were fitted using Eq. 18:

$$F_{abs}=\left[1-\exp\left(-k_{a}t\right)\right]*100\%$$ (18)

### 2.5 Statistical analysis

All statistical analyses were performed using SPSS software package (version 25.0; SPSS Inc., United States) and assessed using Student’s *t*-test. Data with *p* < 0.05 were considered to have a statistically significant difference.

## 3 Results

### 3.1 Characteristics of k_a_, k_10_, k_12_, and k_21_ for drugs with the two-compartment model

The AIC values of 36 IR formulations were determined. All the drugs were more suitable for the two-compartment model because the AIC_2_ values (for the two-compartment model) were smaller than the AIC_1_ values (for the one-compartment model; Table 1). The ranges of k_a_ (0.210–1.726 h^−1^), k_12_ (0.044–0.847 h^−1^), k_21_ (0.010–0.451 h^−1^), and k_10_ (0.012–1.003 h^−1^) were estimated. Interestingly, the sum of k_12_ and k_10_ was less than the value of k_a_ for all drugs ((k_a_ > k_12_ + k_10_; Table 1). Additionally, the values of k_a_ and k_12_ were both higher than the values of k_21_ for all drugs (k_a_ > k_12_ > k_21_; Table 1). The mean values of k_10_ were significantly higher than that of k_21_ (^*^ *p* < 0.05), excepted for a few drugs (e.g., acyclovir, daclatasvir, and levonorgestrel), whose k_10_ values were less than their k_21_ values. These results provided the rationale for setting the available values of k_a_, k_10_, k_12_, and k_21_ for the drugs satisfying the two-compartment model.

**TABLE 1:** The values of k_a_, k_10_, k_12_, and k_21_ of 36 drugs with IR dosage forms estimated using the WinNonlin software in the two-compartment model after oral administrations in human (^***^ *p* < 0.001 vs k_12_, k_21_, k_10_, respectively; ^**^ *p* < 0.01 vs. k_21_; ^*^ *p* < 0.05 vs k_21_ by Student’s t-test).

Drugs	Dosage forms	States	AIC_1_ ^a^	AIC_2_ ^b^	k_a_ (h^−1^)	k_12_ (h^−1^)	k_21_ (h^−1^)	k_10_ (h^−1^)
Abiraterone acetate Wang et al. (2019)	Tablet	Fasting	13.33	−14.36	0.692	0.218	0.116	0.256
Acyclovir Najib et al. (2005)	Suspension	Fasting	−7.985	−48.56	0.604	0.559	0.031	0.012
Azithromycin Chen et al. (2006)	Tablet	Fasting	11.59	1.397	0.467	0.284	0.055	0.133
Benazepril Rezk and Badr. (2014)	Capsule	Fasting	16.75	−1.237	1.468	0.656	0.045	0.769
Bupropion Parekh et al. (2012)	Tablet	Fed	38.16	−2.913	0.260	0.194	0.011	0.049
Candesartan cilexetil Patel et al. (2017)	Tablet	Fasting	48.06	−10.54	0.400	0.116	0.105	0.252
Captopril Rezende et al. (2007)	Tablet	Fasting	8.377	−62.35	0.854	0.333	0.112	0.490
Celecoxib Park et al. (2012)	Capsule	Fasting	5.855	−18.46	0.342	0.175	0.010	0.166
Ciprofloxacin Choudhury et al. (2017)	Tablet	Fasting	10.64	2.574	0.448	0.044	0.019	0.392
Clopidogrel McGregor (2016)	Tablet	Fasting	39.83	−13.67	1.216	0.163	0.061	0.982
Daclatasvir Abdallah et al. (2018)	Tablet	Fasting	9.363	−7.086	0.864	0.506	0.246	0.168
Domperidone Wang et al. (2020)	Tablet	Fasting	46.31	42.33	1.726	0.847	0.451	0.502
Drotaverine Vancea et al. (2014)	Tablet	Fasting	22.72	−26.80	0.574	0.165	0.076	0.406
Glibenclamide Albu et al. (2007)	Tablet	—	32.72	28.62	0.535	0.436	0.012	0.096
Hydrochlorothiazide Kumar et al. (2019)	Tablet	Fasting	17.74	−42.12	0.527	0.168	0.092	0.145
Isradipine Park et al. (2009)	Capsule	Fasting	−4.427	−8.443	0.326	0.153	0.050	0.168
Itraconazole Rhim et al. (2009)	Tablet	Fasting	24.32	−41.14	0.340	0.183	0.063	0.120
Lacidipine Chen et al. (2018)	Tablet	Fasting	9.327	−10.19	0.842	0.377	0.046	0.385
Lercanidipine hydrochloride Li et al. (2016)	Tablet	Fasting	10.99	−9.762	0.649	0.180	0.075	0.438
Levonorgestrel Zhao et al. (2008)	Tablet	Fasting	30.83	−50.02	0.691	0.434	0.178	0.107
Loratadine Vlase et al. (2007)	Tablet	—	43.95	31.72	0.989	0.402	0.063	0.548
Metformin Cho et al. (2018)	Tablet	Fasting	3.769	−42.28	0.542	0.171	0.021	0.358
Mycophenolate mofetil Zhang et al. (2021)	Tablet	Fed	44.21	16.85	1.013	0.736	0.021	0.247
Naproxen Patel et al. (2012)	Tablet	Fasting	18.08	−12.83	0.242	0.195	0.011	0.034
Olmesartan medoxomil Kumar et al. (2019)	Tablet	Fasting	32.02	29.31	0.505	0.160	0.107	0.306
Oseltamivir phosphate Gupta et al. (2013)	Capsule	Fed	30.85	−44.04	0.615	0.153	0.089	0.443
Quinapril Sora et al. (2009)	Tablet	Fasting	13.12	−49.97	0.583	0.053	0.027	0.492
Repaglinide Cho et al. (2018)	Tablet	Fasting	38.56	34.54	1.396	0.314	0.203	1.003
Rilpivirine Gupta et al. (2015)	Tablet	Fed	1.273	−24.65	0.210	0.130	0.051	0.036
Rosuvastatin Zaid et al. (2016)	Tablet	Fasting	15.52	−28.49	0.438	0.117	0.064	0.186
Silodosin Shah and Shrivastav, (2018)	Capsule	Fasting	28.78	−24.60	0.599	0.193	0.073	0.388
Simvastatin Apostolou et al. (2008)	Tablet	—	48.79	−41.32	1.023	0.201	0.161	0.178
Telmisartan Oh et al. (2017)	Tablet	Fasting	9.601	−38.47	0.582	0.255	0.067	0.132
Tenofovir disoproxil fumarate Lu et al. (2019)	Tablet	Fasting	19.61	−45.77	1.089	0.703	0.211	0.211
Terbinafine Bhadoriya et al. (2019)	Tablet	Fasting	19.12	−30.09	0.703	0.252	0.133	0.373
Ticagrelor Chae et al. (2019)	Tablet	—	31.86	−20.44	0.570	0.208	0.063	0.331
Mean	NA^c^	NA	NA	NA	0.692^***^	0.290^**^	0.089	0.314^*^

Notes:

^a^ AIC_1_: AIC, values for the one-compartment model.

^b^ AIC_2_: AIC, values for the two-compartment model.

^c^ NA: not applicable.

### 3.2 Assessing the accuracy of k_a_ estimated using the direct method with the setting parameters

**FIGURE 2:**
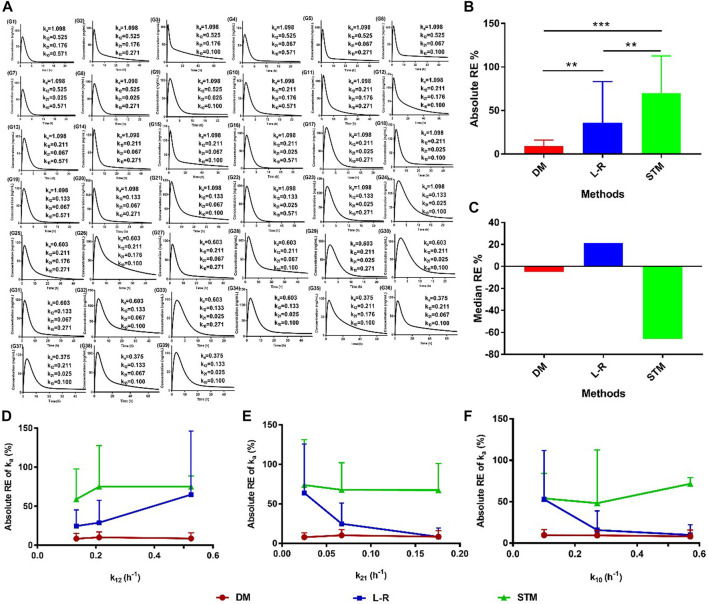
Accuracy of the k_a_ estimated using the direct method with the setting parameters. **(A)** Plasma drug concentration–time profiles of the setting groups; **(B)** absolute values and **(C)** median values of RE for the k_a_ values estimated using different methods. Absolute RE of k_a_ with changes in **(D)** k_12_, **(E)** k_21_, and **(F)** k_10_ estimated using different methods. Data are presented as mean ± standard deviation, ^**^ *p* < 0.01 and ^***^ *p* < 0.001. DM: direct method; L-R: Loo-Riegelman method; RE: relative error; STM: statistical moment method.

To investigate the accuracy and sensitivity of the direct method, the high, medium, and low values of k_a_, k_12_, k_21_, and k_10_ were set according to previous reports (Table 1). The setting values of k_a_ were 1.098, 0.603, and 0.375 h^−1^; the setting values of k_12_ were 0.525, 0.211, and 0.133 h^−1^; the setting values of k_21_ were 0.176, 0.067, and 0.025 h^−1^; the setting values of k_10_ were 0.571, 0.271, and 0.100 h^−1^, respectively (Table 2). Thirty-nine groups were finally obtained with the combination of the values of k_a_, k_12_, k_21_, and k_10_ based on the relationships among them (k_a_ > k_12_ + k_10_, k_a_ > k_12_ > k_21_). All groups satisfied the two-compartment model (AIC_1_ > AIC_2_; Table 2). The values of T_max_, k_max_, and τ were obtained from the drug concentration−time curves of the corresponding group (Figure 2A), which showed that the T_max_ increased following a decrease in k_a_. The values of k_a_ were then estimated using the direct method, Loo-Riegelman method, and statistical moment method. The RE of the k_a_ estimated using the direct method had both positive and negative values when compared with the setting k_a_ (i.e., the true k_a_ value), the values of which were less than 20% in most groups. However, all RE values obtained using the Loo-Riegelman method were positive, wherein estimated k_a_ > true k_a_. On the contrary, most of the RE values obtained using the statistical moment method were negative, wherein estimated k_a_ < true k_a_.

**TABLE 2:** The k_a_ values estimated using the different methods with the setting data (39 groups).

True k_a_ (h^−1^)	k_12_ (h^−1^)	k_21_ (h^−1^)	k_10_ (h^−1^)	AIC_1_ ^a^	AIC_2_ ^b^	T_max_ (h)	τ (h)	k_max_ (h^−1^)	Estimation k_a_ (h^−1^)					
									DM^c^	RE%	L-R^d^	RE%	STM^e^	RE%
1.098	0.525	0.176	0.571	65.15	12.18	0.9	2.5	0.507	1.173	6.80	1.110	1.09	NA^f^	—
	0.525	0.176	0.271	26.81	−26.05	1.1	2.7	0.360	0.947	−13.8	1.166	6.23	0.528	−51.9
	0.525	0.176	0.100	16.97	−104.6	1.3	2.8	0.261	1.144	4.20	1.500	36.6	0.145	−86.8
	0.525	0.067	0.571	47.68	−14.69	0.9	3	0.627	1.358	23.7	1.231	12.1	0.366	−66.7
	0.525	0.067	0.271	43.12	−43.07	1.1	3.1	0.476	1.104	0.57	1.482	35.0	0.246	−77.6
	0.525	0.067	0.100	42.83	−226.9	1.2	3.2	0.373	1.125	2.47	2.287	108	NA	—
	0.525	0.025	0.571	49.35	12.17	0.9	3.5	0.722	1.264	15.1	1.515	38.0	0.203	−81.5
	0.525	0.025	0.271	43.60	−0.498	1.1	3.7	0.566	1.021	−7.01	2.056	87.2	NA	—
	0.525	0.025	0.100	41.51	−231.7	1.2	3.9	0.457	1.035	−5.71	3.946	259	0.149	−86.4
	0.211	0.176	0.571	40.95	21.88	1.1	3.3	0.484	1.120	1.99	1.099	0.05	0.338	−69.2
	0.211	0.176	0.271	23.41	−214.9	1.4	3.5	0.291	1.008	−8.24	1.108	0.89	0.336	−69.4
	0.211	0.176	0.100	0.626	−199.7	1.7	3.7	0.164	1.014	−7.64	1.201	9.36	2.560	133
	0.211	0.067	0.571	42.73	33.98	1.1	3.8	0.559	1.043	−4.98	1.133	3.18	NA	—
	0.211	0.067	0.271	39.15	−215.7	1.4	4	0.353	0.919	−16.3	1.269	15.6	0.306	−72.1
	0.211	0.067	0.100	31.53	−202.8	1.6	4.1	0.220	0.994	−9.43	1.508	37.4	0.065	−94.1
	0.211	0.025	0.571	48.90	−83.11	1.1	4.4	0.620	0.997	−9.16	1.252	14.0	0.243	−77.9
	0.211	0.025	0.271	48.24	−215.1	1.3	4.7	0.403	1.030	−6.15	1.451	32.2	0.143	−87.0
	0.211	0.025	0.100	37.21	−72.15	1.6	4.8	0.259	0.941	−14.3	2.137	94.6	0.169	−84.6
	0.133	0.067	0.571	41.41	24.34	1.1	4.2	0.543	1.107	0.83	1.117	1.69	0.298	−72.9
	0.133	0.067	0.271	38.07	−5.887	1.5	4.4	0.320	0.887	−19.2	1.155	5.19	0.303	−72.4
	0.133	0.067	0.100	23.22	−118.2	1.8	4.5	0.176	0.941	−14.3	1.336	21.7	0.519	−52.7
	0.133	0.025	0.571	40.97	−49.38	1.1	4.8	0.590	1.069	−2.63	1.192	8.53	0.430	−60.8
	0.133	0.025	0.271	38.34	−43.25	1.4	5.1	0.354	1.002	−8.77	1.313	19.6	NA	-
	0.133	0.025	0.100	32.78	11.05	1.8	5.2	0.202	0.907	−17.4	1.737	58.2	0.382	−65.2
0.603	0.211	0.176	0.271	8.731	−23.04	2	5	0.211	0.684	13.5	0.609	0.95	0.485	−19.6
	0.211	0.176	0.100	−17.30	−72.15	2.5	5.3	0.114	0.669	11.0	0.660	9.44	0.194	−67.8
	0.211	0.067	0.271	37.30	−14.10	1.9	5.5	0.273	0.654	8.49	0.661	9.68	0.162	−73.1
	0.211	0.067	0.100	21.91	−35.52	2.4	5.8	0.171	0.573	−5.06	0.825	36.8	0.402	−33.3
	0.211	0.025	0.271	38.10	−50.63	1.9	6.4	0.325	0.596	−1.12	0.793	31.6	2.065	243
	0.211	0.025	0.100	34.48	−73.74	2.3	6.8	0.218	0.560	−7.08	1.152	91.0	0.313	−48.1
	0.133	0.067	0.271	35.48	−40.65	2.1	6.2	0.259	0.572	−5.19	0.635	5.24	1.527	153
	0.133	0.067	0.100	13.57	−211.7	2.7	6.5	0.144	0.531	−12.0	0.733	21.5	0.180	−70.1
	0.133	0.025	0.271	37.42	−487.6	2	7.2	0.300	0.596	−1.20	0.719	19.3	0.562	−6.8
	0.133	0.025	0.100	27.53	−240.3	2.6	7.6	0.177	0.529	−12.3	0.944	56.6	0.393	−34.8
0.375	0.211	0.176	0.100	38.82	−39.54	3.5	7.1	0.077	0.482	28.6	0.412	9.79	0.529	41.1
	0.211	0.067	0.100	8.348	−81.50	3.1	7.4	0.127	0.454	21.1	0.515	37.2	0.266	−29.1
	0.211	0.025	0.100	26.34	−198.9	3	8.5	0.173	0.409	9.00	0.714	90.4	0.242	−35.5
	0.133	0.067	0.100	1.883	−15.28	3.5	8.6	0.113	0.408	8.80	0.457	21.9	0.422	12.5
	0.133	0.025	0.100	21.38	−92.82	3.4	9.9	0.148	0.375	0.09	0.587	56.5	0.194	−48.3

Notes:

a AIC_1_: AIC, values for the one-compartment model.

b AIC_2_: AIC, values for the two-compartment model.

c DM: direct method.

d L-R: Loo-Riegelman method.

e STM: statistical moment method.

f NA: MAT in negative.

The absolute values of RE were calculated, and the data are shown in Figure 2B. The absolute RE of k_a_ estimated using the direct method was significantly less than that estimated using either the statistical moment method (^**^ *p* < 0.01) or the Loo-Riegelman method (^***^ *p* < 0.001). The absolute RE of k_a_ estimated using the Loo-Riegelman method was significantly less than that estimated using the statistical moment method (^**^ *p* < 0.01). The median RE of k_a_ estimated using the direct method (−4.98%) was better than that estimated using the Loo-Riegelman method (21.5%) and the statistical moment method (−65.9%; Figure 2C). The accuracy of k_a_ estimated using the direct method was not affected by changes in k_12_, k_21_, and k_10_, which also demonstrated excellent accuracy when compared with that estimated using the Loo-Riegelman method and the statistical moment method (Figures 2D–F). Therefore, the direct method yielded a more accurate value and did not require the determination of k_12_, k_21_, and k_10_ from intravenous PK measurements.

### 3.3 Validation of the direct method in human PK studies

**FIGURE 3:**
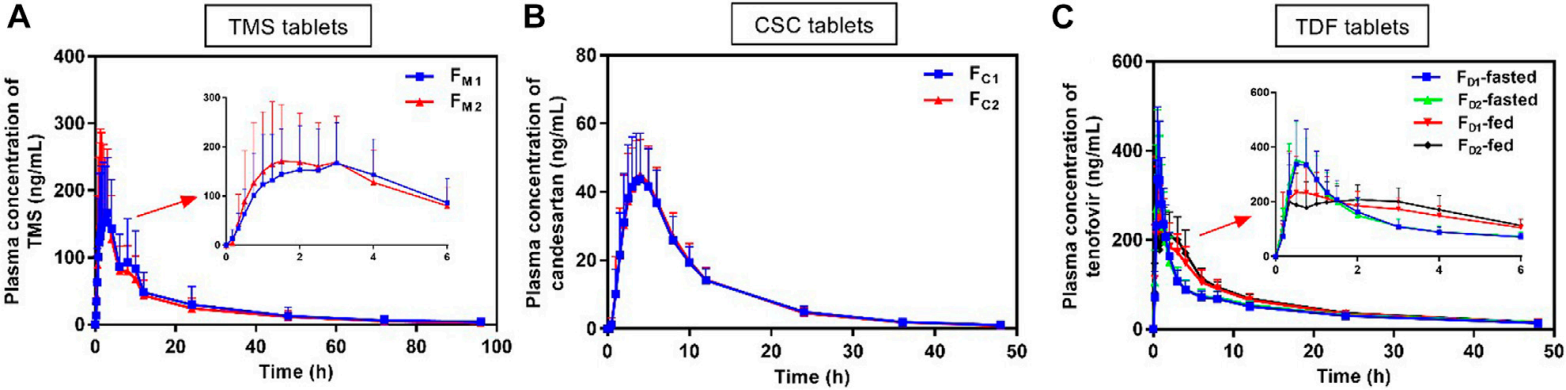
Mean plasma concentration *versus* time profiles of **(A)** TMS, **(B)** candesartan (metabolite of CSC), and **(C)** tenofovir (metabolite of TDF) obtained after the oral administration of TMS (*n* = 26), CSC (*n* = 24), and TDF tablets (*n* = 24) in humans. Data are presented as mean ± standard deviation. CSC: candesartan cilexetil; TDF: tenofovir disoproxil fumarate; TMS: telmisartan.

The mean plasma drug concentration–time curves of TMS, candesartan, and tenofovir were obtained from PK evaluation in human (Figure 3). The PK parameters are listed in Table 3.

**TABLE 3:** PK parameters of TMS, candesartan (metabolite of CSC), tenofovir (metabolite of TDF) following administration of single dose of TMS (*n* = 26), CSC (*n* = 24) and TDF tablets (*n* = 24) in the fasted or/and fed state, respectively. Data are presented as mean ± standard deviation, ^*^ *p* < 0.05 vs. the same formulation in the fasted state.

Drugs-states	Formulations	C_max_ (ng/mL)	AUC_0-t_ (h·ng/mL)	AUC_0-∞_ (h·ng/mL)	T_max_ (h)	t_1/2_ (h)
TMS tablets-Fasted	F_M1_	187.57 ± 98.83	2563.31 ± 1794.97	2691.41 ± 1914.39	2.62 ± 0.91	20.79 ± 6.99
	F_M2_	206.81 ± 119.41	2299.54 ± 1324.28	2406.63 ± 1410.20	2.34 ± 0.88	20.95 ± 8.45
CSC tablets-Fasted	F_C1_	46.77 ± 14.51	501.20 ± 121.31	516.55 ± 130.08	4.01 ± 1.03	9.21 ± 3.92
	F_C2_	48.28 ± 11.98	503.69 ± 109.05	514.33 ± 110.82	3.77 ± 0.80	8.82 ± 1.68
TDF tablets-Fasted	F_D1_	391.54 ± 130.91	2239.18 ± 482.78	2615.50 ± 584.69	0.78 ± 0.46	18.50 ± 2.30
	F_D2_	398.85 ± 113.10	2315.77 ± 469.52	2709.84 ± 560.22	0.76 ± 0.50	14.46 ± 2.68
TDF tablets-Fed	F_D1_	319.56 ± 115.77^*^	2648.72 ± 531.53	3037.13 ± 633.74	1.03 ± 0.91	16.81 ± 2.37
	F_D2_	289.93 ± 72.50^*^	2745.78 ± 297.12	3107.13 ± 344.37	1.29 ± 1.02^*^	16.46 ± 1.92

The mean plasma concentrations of F_M2_ were higher than those of F_M1_ over a period of 0.5–3.0 h after oral administration (Figure 3A), and the C_max_ of F_M2_ was higher than that of F_M1_ (Table 3). Overall, the plasma drug concentration–time profiles (Figure 3B) and the PK parameters (Table 3) of F_C1_ and F_C2_ were similar. The C_max_ of tenofovir in the fed state was significantly lower than that of tenofovir in the fasted state for both F_D1_ and F_D2_ (^*^ *p* < 0.05; Figure 3C; Table 3), and the T_max_ of tenofovir in the fed state was also larger than that of tenofovir in the fasted state (^*^ *p* < 0.05 for F_D2_). The three model drugs with different T_max_ values (0.5–4.0 h) represented low, medium, and high absorption rates of the IR dosage forms.

The k_a_ values of the TMS, CSC, and TDF tablets were estimated using different methods. Data of intravenous PK parameters of TMS were obtained from a previously published report (Stangier et al., 2000) and were used to estimate k_a_ using the Loo-Riegelman method. However, it was difficult to acquire the *in vivo* data of CSC, TDF, and their respective metabolites (candesartan, tenofovir) after intravenous administration. The k_a_ value for F_M2_ estimated using the direct method was higher than that of F_M1_ estimated using the same method. These values had a consistent trend with the estimation of k_a_ using the Loo-Riegelman method, but it had a contrary trend to the estimation of k_a_ using the statistical moment method (Table 4). k_a_ estimated using the direct method for F_C1_ was similar to that of F_C2_, whereas k_a_ estimated using the statistical moment method of F_C1_ was higher than that of F_C2_. The estimated k_a_ of both F_D1_ and F_D2_ in the fasted state was higher than those of F_D1_ and F_D2_ in the fed state (^*^ *p* < 0.05 for F_D2_). The k_max_ values of both F_D1_ and F_D2_ in the fasted state were also higher than that of F_D1_ and F_D2_ in the fed state (^*^ *p* < 0.05). Moreover, the k_a_ value of F_D1_ was consistent with that of F_D2_ estimated using the direct method in the same state. This finding was contrary to that obtained using the statistical moment method, which yielded the k_a_ value of F_D1_ that was higher than that of F_D2_.

**TABLE 4:** The k_a_ values estimated using the different method for the TMS, CSC, TDF tablets in the fasted or/and fed state. Data are presented as mean ± standard deviation, ^*^ *p* < 0.05 vs. k_a_ value of the same formulation estimated using the direct method in the fasted state.

Drugs-states	Formulations	τ (h)	k_max_ (h^−1^)	Estimation k_a_ (h^−1^)		
				Direct method	Statistical moment method	Loo-Riegelman method
TMS tablets-fasted	F_M1_	6.88 ± 3.31	0.35 ± 0.12	0.486 ± 0.314	0.203 ± 0.145	0.677 ± 0.363
	F_M2_	6.13 ± 2.97	0.33 ± 0.14	0.588 ± 0.381	0.190 ± 0.121	0.778 ± 0.331
CSC tablets-fasted	F_C1_	7.64 ± 1.55	0.20 ± 0.03	0.273 ± 0.132	0.819 ± 0.486	NA^a^
	F_C2_	7.53 ± 2.25	0.20 ± 0.04	0.280 ± 0.125	0.671 ± 0.318	NA
TDF tablets-fasted	F_D1_	1.83 ± 0.69	1.07 ± 0.48	1.459 ± 0.659	0.666 ± 0.563	NA
	F_D2_	1.67 ± 0.68	1.04 ± 0.38	1.499 ± 0.562	0.455 ± 0.445	NA
TDF tablets-fed	F_D1_	2.26 ± 1.31	0.57 ± 0.40^*^	1.142 ± 0.616	0.715 ± 0.303	NA
	F_D2_	2.42 ± 1.45	0.64 ± 0.29^*^	1.047 ± 0.613^*^	0.590 ± 0.477	NA

Notes:

^a^ NA: not applicable, as which has no intravenous PK data.

**FIGURE 4:**
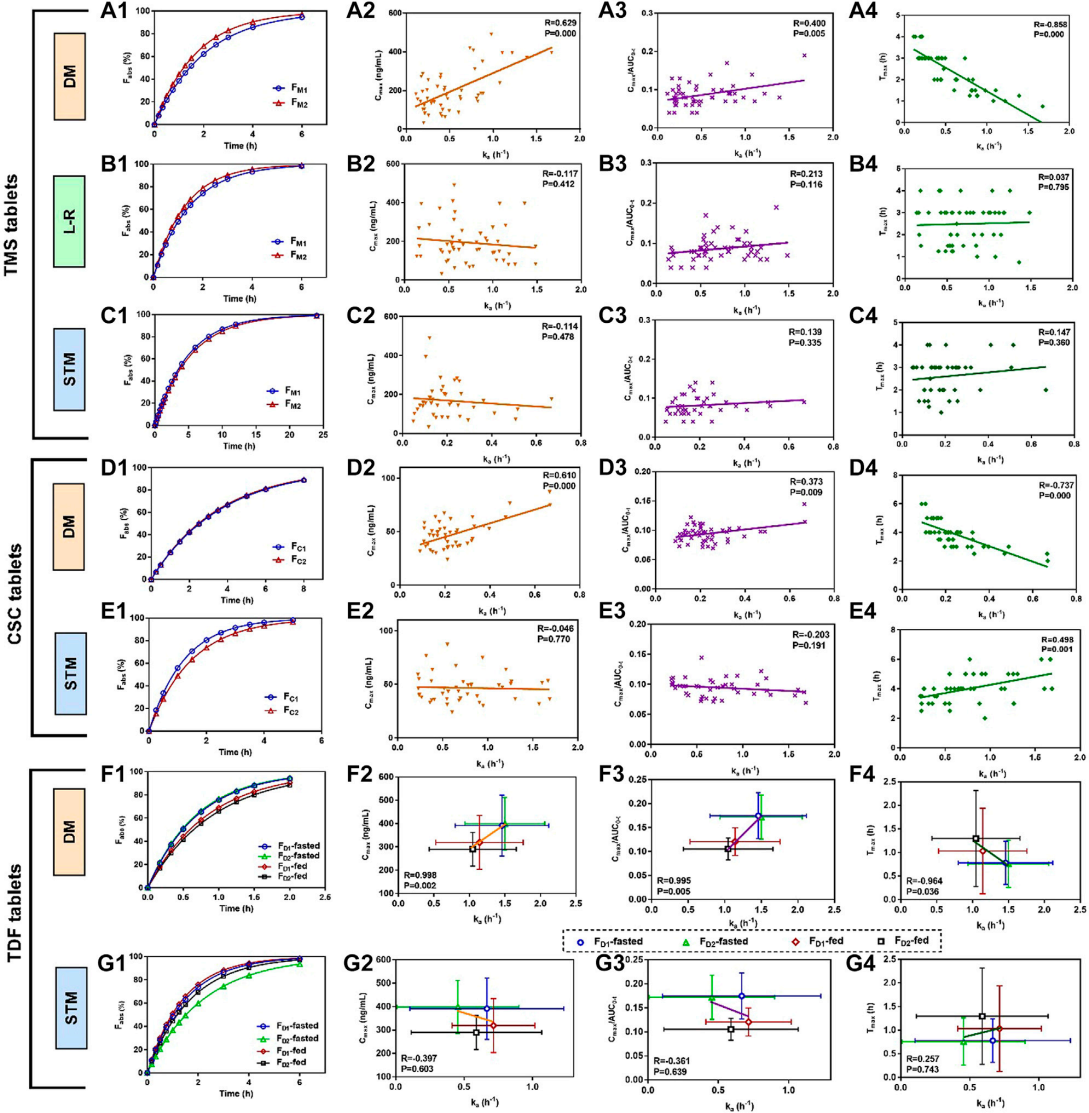
Mean absorbed fraction *versus* time profiles of TMS tablets, CSC tablets, and TDF tablets and correlations between estimated k_a_ values and the other PK parameters that reflected the absorption properties of the drugs *in vivo* (T_max_, C_max_ and C_max_/AUC_0-t_). **(A1)** Mean absorbed profiles of TMS tablets estimated using the DM, and the correlations between the values of k_a_ and **(A2)** C_max_, **(A3)** C_max_/AUC_0-t_, **(A4)** T_max_; **(B1)** mean absorbed profiles estimated using the L-R method, and the correlations between the values of k_a_ and **(B2)** C_max_, **(B3)** C_max_/AUC_0-t_, **(B4)** T_max_; **(C1)** mean absorbed profiles estimated using the STM, and the correlations between the values of k_a_ and **(C2)** C_max_, **(C3)** C_max_/AUC_0-t_, **(C4)** T_max_; **(D1)** mean absorbed profiles of CSC tablets estimated using the DM, and the correlations between the values of k_a_ and **(D2)** C_max_, **(D3)** C_ma_x/AUC_0-t_, **(D4)** T_max_; **(E1)** mean absorbed profiles estimated using the STM, and the correlations between the values of k_a_ and **(E2)** C_max_, **(E3)** C_max_/AUC_0-t_ and **(E4)** T_max_. **(F1)** Mean absorbed profiles of TDF tablets obtained using the DM, and the correlations between the values of k_a_ and **(F2)** C_max_, **(F3)** C_max_/AUC_0-t_, **(F4)** T_max_; **(G1)** mean absorbed fraction *versus* time profiles of TDF tablets obtained using the STM, and the correlations between the values of k_a_ and **(G2)** C_max_, **(G3)** C_max_/AUC_0-t_, and **(G4)** T_max_. Data of the correlations for TDF tablets are presented as mean ± standard deviation. All correlations were investigated using Pearson’s correlation analysis (*p* < 0.05 indicates good correlation). CSC: candesartan cilexetil; DM: direct method; L-R method: Loo-Riegelman method; PK: pharmacokinetic; STM: statistical moment data; TDF: tenofovir disoproxil fumarate; TMS: telmisartan.

The mean absorbed fraction-time profiles of TMS tablets showed that the absorbed fraction of F_M2_ was faster than those of F_M1_ using the direct method and the Loo-Riegelman method within the first 4 h (Figures 4A1, B1), which was consistent with the mean plasma drug concentration-time profiles (Figure 3A) and C_max_ value of TMS (Table 3). However, the absorption profiles of F_M1_ and F_M2_ had nearly overlapped when estimated using the statistical moment method (Figure 4C1), which was inconsistent with their *in vivo* experimental data. The values of k_a_ estimated using the direct method were positively correlated with both C_max_ and C_max_/AUC_0-t_ (correlation coefficient (R) > 0.4, *p* < 0.01; Figures 4A2, A3) and negatively correlated with T_max_ (R = −0.858, *p* < 0.001; Figure 4A4) as observed in Pearson’s correlation analysis. However, the k_a_ estimated using the Loo-Riegelman method (Figures 4B2–B4) and the statistical moment method (Figures 4C2–C4) demonstrated only slight correlation with these parameters (*p* > 0.1).

The similarity in the estimated k_a_ between F_C1_ and F_C2_ led to nearly overlapped absorbed fraction-time profiles using the direct method (Figure 4D1). The estimated k_a_ values were positively correlated with both C_max_ and C_max_/AUC_0-t_ (*p* < 0.01; Figures 4D2, D3) and negatively correlated with T_max_ (*p* < 0.001; Figure 4D4). However, these two profiles were not similar when estimated using the statistical moment method (Figure 4E1), which were inconsistent with their *in vivo* performance (Figure 3B). The k_a_ estimated using the statistical moment method also showed only slight correlation with both C_max_ and C_max_/AUC_0-t_ (Figures 4E2, E3), while it was positively correlated with T_max_ (Figure 4E4).

The mean absorbed fraction-time profiles of the TDF tablets were obtained after the k_a_ values were estimated using both the direct method (Figure 4F1) and the statistical moment method (Figure 4G1). The absorptions of TDF in both formulations (F_D1_ and F_D2_) in the fasted state were higher than that in the fed state when assessed using the direct method. The estimated k_a_ values for F_D1_ and F_D2_ were strongly correlated with the corresponding average values of C_max_, C_max_/AUC_0-t_, and T_max_ in both the fed and fasted states (R > 0.96, *p* < 0.05; Figures 4F2–F4). However, data from the absorption curves were inconsistent with the *in vivo* concentration data (Figure 3C) when assessed using the statistical moment method (Figure 4G1). Furthermore, the k_a_ estimated using the statistical moment method had only slight correlations with C_max_, C_max_/AUC_0-t_, and T_max_ (*p* > 0.6; Figures 4G2–G4).

## 4 Discussion

The k_a_, k_12_, k_21_, and k_10_ values of drugs with the two-compartment model have shown variation owing to their physicochemical properties and dosage form (Byron and Notari, 1976), but the relationships between these parameters have not been reported. The accuracies of the estimated k_a_, k_12_, k_21_, and k_10_ values for the IR formulations of drugs are higher than those for the extended-release formulations because the former is affected at a lesser rate by the rate of dissolution *in vivo* (Franek et al., 2015). In this case, 36 IR dosage forms with different T_max_ (0.75–4.0 h) and t_1/2_ (1.2–52.8 h) values, as well as satisfying the two-compartment model, were used to estimate the k_a_, k_12_, k_21_, and k_10_ values (Table 1), mainly for investigating the relationships between these parameters. In theory, the value of k_12_ should be higher than that of k_21_ (k_12_ > k_21_) because of the dynamics of drug distribution from the central compartment to the peripheral compartment. Meanwhile, the absorption rate of a drug needs to be greater than the sum of the distribution and elimination rates (k_a_ > k_12_ + k_10_), so that the concentration of a drug can be determined in the plasma after extravascular administration. Elucidating the relationships between these parameters could circumvent any void in setting data for investigations using the direct method. However, the k_a_, k_12_, k_21_, and k_10_ values of 36 IR dosage forms were assessed only by preliminary quantification to observe their relationships using WinNonlin software (built-in residual method). As expected, the k_a_ values of TMS, CSC, and TDF estimated using WinNonlin software were different from the values of k_a_ calculated using the direct method (Tables 1, 4).

The range of T_max_ for all setting groups was 0.5–4.0 h (Table 2), which was representative of the *in vivo* performance of most of the IR dosage forms in practice. The k_a_ estimated using the direct method was evidently affected by k_max_, T_max_, and τ values (Eq. 6), and the negative correlation between k_max_ and T_max_ (or τ) could ensure that the estimated k_a_ was accurate and independent of the changes in k_12_, k_21_, and k_10_ (Figures 2D–F). The statistical moment method, as a non-compartmental method, should be non-sensitive to the changes in compartmental parameters (i.e., k_12_, k_21_, and k_10_). However, the most values of k_a_ estimated using the statistical moment method had low levels (Table 2) because small values of k_T_ were obtained from the terminal sampling point (Riegelman and Collier, 1980). The estimated values of k_a_ were undoubtedly and sensitively affected by k_12_, k_21_, and k_10_ values when applying the Loo-Riegelman method (Figures 2D–F), according to Eqs 14, 15 (Byron and Notari, 1976; Zeng et al., 2020). However, all the estimated values of k_a_ were higher than the true values of k_a_, which might have been attributed to the difference in the number of time points in the unabsorbed fraction (1–F_abs_%) that were fitted in the linear regression analysis. Moreover, the mean absolute RE of the k_a_ estimated using the Loo-Riegelman method had a relatively large value because of a few outliers (RE > 100%) that negatively affected the fitting precision, but it also had a better estimating accuracy than that of the statistical moment method (Figures 2B, C).

The three model drugs, whose T_max_ (0.5–4.0 h) values were different, were selected to explore the accuracy and scopes of the direct method in practice (Oh et al., 2017; Patel et al., 2017; Lu et al., 2019). The empirical k_a_ values of these drugs could not be obtained from previously reported studies. Therefore, the relationships between the absorption rate and the PK data were investigated to indirectly verify the accuracy of the direct method. Generally, the high absorption rate of the drugs showed a large C_max_ and a short T_max_ (Han et al., 2018). The values of C_max_ and C_max_/AUC_0-t_ represented the *in vivo* exposure of the drugs, which were also related to the k_a_ values (Tozer et al., 1996). The k_a_ values of the three model drugs estimated using the direct method were positively correlated with the *in vivo* exposure of TMS (Figures 4A2, A3), CSC (Figures 4D2, D3), and TDF (Figures 4F2, F3), which might be advantageous in predicting the *in vivo* exposure of the different formulations. Negative correlations were observed between k_a_ and T_max_ (Figures 4A4, 4D4, 4F4), which were consistent with previous literature results (Han et al., 2018). However, both the Loo-Riegelman method (used only for TMS) and the statistical moment method failed to establish the correlation between the estimated k_a_ and their C_max_, C_max_/AUC_0-t_, and T_max_ values. The k_a_ of CSC estimated using the statistical moment method was positively correlated with T_max_, which was contrary to the literature precedent (Han et al., 2018).

The PK parameters of drugs, including C_max_, AUC_0-t_, T_max_, and k_a_, are generally affected by the intake of high-fat foods (Winter et al., 2013). In this study, the decreased k_a_ and C_max_ values and prolonged T_max_ values of TDF in the fed states were compared to those in the fasted state. The similar *in vivo* results of TDF between the fed and fasted states were consistent with that reported in a previous study (Lu et al., 2013). A difference in the estimated k_a_ of TDF was observed between the fed and fasted states when assessed using the direct method (Table 4), and linear correlations with C_max_, C_max_/AUC_0-t_, and T_max_ values were observed (Figures 4F2–F4). On the contrary, the statistical moment method failed to produce a difference in the estimated k_a_ between the fed and fasted states, and no correlations were observed between the estimated k_a_ and their C_max_, C_max_/AUC_0-t_, and T_max_ values (Figures 4G2–G4). Therefore, these results corroborated that the direct method was sensitive and accurate when estimating k_a_ for applications related to PK evaluations.

Although the absorption of a drug after oral administration was terminated at a finite time point after T_max_ in a previous study (Macheras, 2019), the exact endpoint was unclear. In this study, τ represented the endpoint of the post-absorption phase in the PK profiles, at which the absorption process had finished. The values of τ for TMS, CSC, and TDF tablets were obtained in the fed and/or fasted states (Table 4). The average values of F_abs_ for these drugs exceeded 90% at the mean value of τ (Figures 4A1, 4D1, 4F1), which verified the inference of the direct method.

As the accuracies of k_max_, τ, and T_max_ greatly affected the estimation of k_a_, sufficient sampling points in PK studies might be needed to obtain accurate values of k_max_, τ, and T_max_. In the present study, the sampling points for PK studies of the three model drugs in humans were designed as conventional sampling points (such as 0.17 h, 0.33 h, 0.5 h, 1 h, … ), rather than sampling points with intervals of 0.1 h for the setting data. The conventional points did not significantly affect the calculation of k_a_, demonstrating that the direct method was highly feasible for estimating the absorption rate of drugs in practical applications. However, more drugs fitting with the two-compartment PK model should be evaluated in further studies to verify the accuracy and applicability of the direct method.

## 5 Conclusion

In this study, the direct method was developed and used for estimating the k_a_ value of a drug with the two-compartment model using the equation $T_{\max}=\frac{\ln k_{a}-\ln\frac{{\tau *k}_{\max}}{\tau -T_{\max}}}{k_{a}-\frac{{\tau *k}_{\max}}{\tau -T_{\max}}}$ , wherein the values of T_max_, k_max_, and τ were readily obtained from the plasma drug concentration–time curves after extravascular administration. The k_a_ estimated using the direct method with the setting data had satisfactory accuracy compared with that obtained using both the Loo-Riegelman method and the statistical moment method. The k_a_ values of three model drugs (TMS, CSC, and TDF) were estimated by the direct method, which was consistent with the corresponding PK profiles. From these calculations, good correlations were established between the k_a_ values and other PK parameters that reflected the *in vivo* absorption of the drugs. These results substantiated the accuracy of the direct method in estimating the absorption rate of a drug, which is beneficial in practical applications where intravenous PK data cannot be obtained. The direct method is expected to provide valuable support for PK evaluation and IVIVC establishment.

## Data Availability

The raw data supporting the conclusion of this article will be made available by the authors, without undue reservation.
